# Recent Advancements in the Synthesis of Ultra-High Molecular Weight Polyethylene via Late Transition Metal Catalysts

**DOI:** 10.3390/polym16121688

**Published:** 2024-06-13

**Authors:** Qiang Yue, Rong Gao, Zhihui Song, Qingqiang Gou

**Affiliations:** Department of Polyethylene, SINOPEC (Beijing) Research Institute of Chemical Industry Co., Ltd., Beijing 100013, China

**Keywords:** late transition metal catalyst, ultra-high molecular weight, ethylene polymerization

## Abstract

Ultra-high molecular weight polyethylenes (UHMWPEs) are significant engineering plastics for their unique properties, such as high impact resistance, abrasion resistance, weatherability, lubricity, and chemical resistance. Consequently, developing a suitable catalyst is vital in facilitating the preparation of UHMWPE. The late transition metal catalysts have emerged as effective catalysts in producing UHMWPE due to their availability, enhanced tolerance to heteroatom groups, active polymerization characteristics, and good copolymerization ability with polar monomers. In this review, we mainly focus on the late transition metal catalysts, summarizing advancements in their application over the past decade. Four key metals (Ni, Pd, Fe, Co) for generating linear or branched UHMWPE will be primarily explored in this manuscript.

## 1. Introduction

Ultra-high molecular weight polyethylene (UHMWPE) commonly denotes linear long-chain polyethylene with a molecular weight higher than 10^6^ g·mol^−1^ [1]. UHMWPE is an excellent class of advanced engineering plastics with unique properties, such as high strength, impact resistance, outstanding abrasive wear resistance, good chemical corrosion resistance, self-lubrication, and biocompatibility [2,3,4]. The applications of UHMWPE span various fields including the national defense and military industry, aerospace, marine engineering, petrochemicals, medical equipment, sports equipment, new energy, new materials, etc. [5,6,7].

Notably, the synthesis of UHMWPE is a challenging task that requires suitable catalysts. The primary commercial catalyst employed for the synthesis of UHMWPE is the Ziegler–Natta (ZN) catalyst [8]. However, transition metal catalysts have distinct advantages over the conventional ZN catalytic system, enabling the production of polymers with precisely controlled molecular weights, fewer entanglements, and narrower molecular weight distributions. In recent years, some highly active and thermally stable early transition metal catalysts have facilitated the preparation of high-performance UHMWPE [9].

However, these oxophilic early metal catalysts cannot tolerate functional groups. So, the early transition metal catalysts cannot be used to synthesize copolymers of ethylene and polar vinyl monomers like alkyl acrylates and vinyl acetate. Copolymerizations of polar monomers with ethylene using more functional group tolerant late metal catalysts potentially offer an attractive alternative for generating the high-performance copolymerized UHMWPE. In addition, the late transition metal catalysts not only retain all the advantages of early transition metal catalysts, but also have additional advantages such as enhanced tolerance to heteroatom groups, active polymerization characteristics, and good copolymerization ability with polar monomers, enabling the production of UHMWPE with required properties [10].

In general, heterogeneous catalytic systems dominate the polyolefin industry due to their superior properties in product morphology control and avoidance of reactor fouling. However, the late transition metal catalysts mainly focus on academic research in the homogeneous catalysis field. The late transition metal catalysts have advantages such as diverse structures, robust stability, and cost-effectiveness. These catalysts for the production of UHMWPE can contribute to sustainable development, reducing the consumption of resources and waste. In recent years, researchers have conducted a series of basic research and application development in the late transition metal catalyst versatile structures, mechanistic studies about homogeneous catalytic ethylene copolymerization [11], supported catalyst technology, and preparation of high-performance UHMWPE with supported catalysts. The final goal is to achieve the application of the late transition metal catalysts in industrial production.

Late transition metal catalysts not only represent an evolution in the field of materials, but also provide a technological advance in the entire range of industries that depend on advanced and high-performance materials. Therefore, the research of late transition metal catalysts has significantly enabled the preparation of high-performance UHMWPE. This review mainly discusses the late transition metal catalysts in the synthesis of UHMWPE.

## 2. Late Transition Metal Catalysts

**Figure 1:**
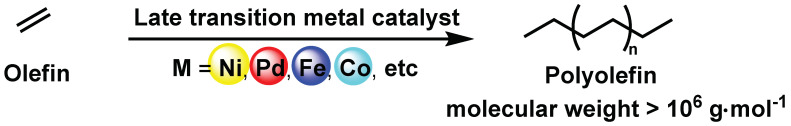
Synthesis of UHMWPE using late transition metal catalysts.

Late transition metal catalysts have strong chain walking ability in ethylene polymerization, providing enormous potential for producing superior UHMWPE. In this section, according to the different central metals, late transition metal catalysts mainly consist of complexes formed using transition metals such as nickel, palladium, iron, cobalt, etc. (Figure 1).

### 2.1. Nickel-Based Catalysts with N,N′ Ligands

**Figure 2:**
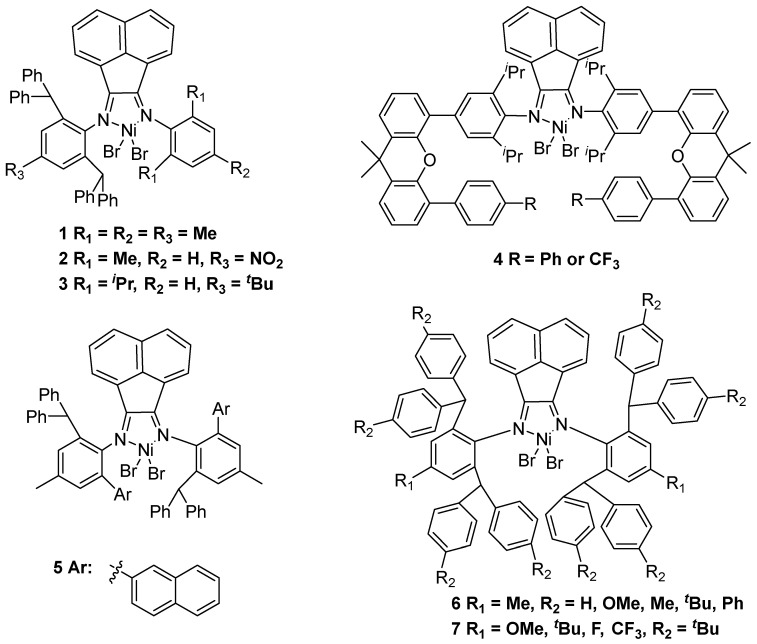
Acenaphthene-based α-diimine nickel catalysts **1**–**7** for ethylene polymerization to UHMWPE.

In 1995, the Brookhart type α-diimine nickel catalyst was first reported as a significant breakthrough contribution in nickel-catalyzed ethylene polymerization [12]. This class of catalysts has high activities along with the ability to produce high molecular weight polyethylene. Notably, some nickel-based catalysts with N,N′ ligands have high thermal stability to afford UHMWPE. In 2011, the Redshaw and Sun group disclosed a series of nickel dibromide complexes with 2,6-dibenzhydryl-N-(2-phenyliminoacenaphthylenylidene)-4-methylbenzenamines to produce UHMWPE using methylaluminoxane (MAO) or diethylaluminum chloride (Et_2_AlCl) as cocatalysts [13]. It should be noted that catalyst **1** affords the highest molecular weight polyethylene with *M*_w_ = 1.04 × 10^6^–1.28 × 10^6^ g·mol^−1^ at 20 °C and 10 bar ethylene pressure (Figure 2). In 2017, the Sun group showed that analogous structure complexes with 1-(2,6-dibenzhydryl-4-nitrophenylimino)-2-(arylimino)acenaphthylene could generate UHMWPE using MAO as a cocatalyst [14]. Moreover, the prepared polyethylene exhibited the highest molecular weight of up to 2.41 × 10^6^ g·mol^−1^ using complex **2** as the catalyst. Subsequently, the same group discovered that the unsymmetrical structure catalyst **3** could afford UHMWPE in ethylene polymerization using modified methylaluminoxane (MMAO) as a cocatalyst [15]. At the same time, the Dai and Chen group reported that α-diimine nickel complexes **4** with acenaphthene backbone could prepare UHMWPE using MAO as a cocatalyst at 20 or 40 °C [16]. It should be noted that the obtained branched UHMWPE with *M*_n_ up to 1.54 × 10^6^ g·mol^−1^ using the nickel catalyst bearing a CF_3_ substituent.

In 2018, the Chen group synthesized unsymmetric α-diimine nickel complexes with acenaphthene structure, and the ability to prepare UHMWPE using MAO as a cocatalyst [17]. The corresponding nickel catalyst **5** was able to generate a polymeric product with a very high molecular weight (*M*_n_ up to 1.80 × 10^6^ g·mol^−1^) at 50 °C. Simultaneously, the Dai group discovered that a series of sterically hindered acenaphthene-based α-diimine nickel complexes **6** could obtain moderate to highly branched (26–71 branches/1000 C) UHMWPE with *M*_w_ up to 4.50 × 10^6^ g·mol^−1^ using Et_2_AlCl as a cocatalyst [18]. Notably, the remote substituent R in the 4-position of the diarylmethyl moiety in these nickel complexes could significantly affect the catalytic properties. In 2022, the Qasim and Behzadi group reported that similar structure nickel complexes could produce UHMWPE using Et_2_AlCl as a cocatalyst [19]. Incredibly, these catalysts have thermal stability at 120 °C to afford UHMWPE with *M*_n_ up to 3.33 × 10^6^ g·mol^−1^ in *n*-heptane solvent at 30 bar of ethylene pressure.

**Figure 3:**
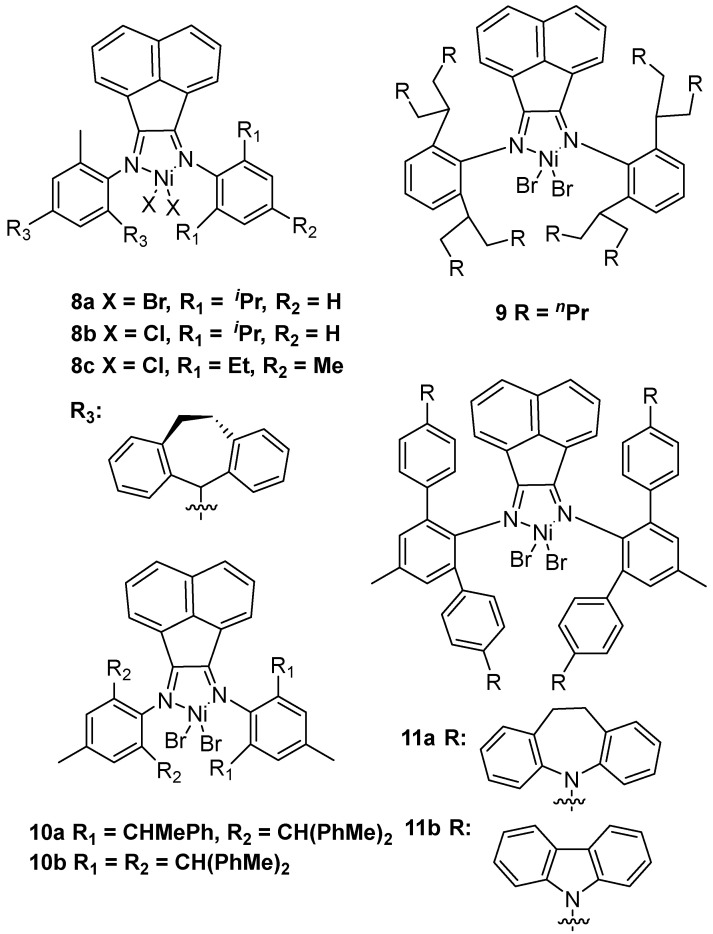
Acenaphthene-based α-diimine nickel catalysts **8**–**11** for ethylene polymerization to UHMWPE.

In 2019, the Sun group achieved a series of unsymmetrical acenaphthene nickel complexes **8** to generate UHMWPE using MAO as a cocatalyst in toluene at 30 °C and 10 bar ethylene pressure (Figure 3) [20]. It should be noted that nickel catalysts with bromide or chloride substituents all could obtain UHMWPE. At the same time, the Dai group used n-propyl substituent α-diimine nickel **9** to afford UHMWPE with *M*n = 1.20 × 10^6^ g·mol^−1^ using MAO as a cocatalyst [21]. Most importantly, the synthesized corresponding polyethylene has excellent elastic properties. Simultaneously, the same group disclosed acenaphthene-based α-diimine nickel complexes **10** with bulky diarylmethyl substituents for producing UHMWPE [22]. Notably, the nickel catalyst **10b** has excellent thermal stability at 80 °C to generate UHMWPE with *M*n up to 1.21 × 10^6^ g·mol^−1^. In 2022, the Jian group used the concerted double-layer steric strategy and the rotation-restricted strategy to design α-diimine nickel catalyst **11** for producing UHMWPE using MAO as a cocatalyst [23]. More importantly, these nickel catalysts could be able to obtain almost linear UHMWPE.

**Figure 4:**
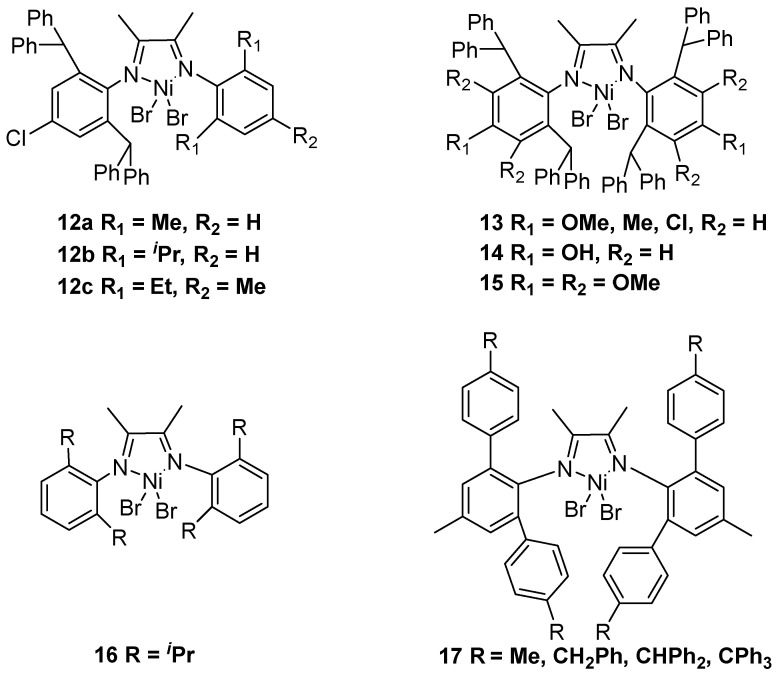
Classical α-diimine nickel catalysts **12–17** for ethylene polymerization to UHMWPE.

Apart from acenaphthene-based α-diimine nickel catalysts, the classical α-diimine nickel catalysts with N,N′ ligands could also generate UHMWPE. In 2013, the Sun group reported a series of unsymmetrical α-diimine nickel bromide complexes **12** to produce UHMWPE using MAO or MMAO as cocatalysts (Figure 4) [24]. When MAO was the cocatalyst, catalyst **12a** could generate UHMWPE with bimodal distributions. In 2016, the Chen group synthesized a range of electron-donating and -withdrawing substituents α-diimine nickel complexes **13** to prepare UHMWPE using MAO or Et_2_AlCl as cocatalysts [25]. Notably, these nickel catalysts show excellent thermal stability at 100 °C to generate UHMWPE with *M*n up to 1.54 × 10^6^ g·mol^−1^.

In 2019, the same group disclosed an analogous structure complex **14** with hydroxy substituent to produce UHMWPE using MAO as a cocatalyst [26]. It should be noted that the corresponding polymer had a significantly increased melting temperature (up to 120 °C) and a greatly decreased number of branching (37 per 1000 carbon atoms). Subsequently, the authors further reported a similar structure complex **15** to obtain UHMWPE in toluene at 100 °C and 9 bar ethylene pressure [27].

In 2020, the Li and Wang group achieved the simple α-diimine nickel complex **16** that was supported with polyhedral oligomeric silsesquioxane (POSS) material to obtain UHMWPE using MMAO as a cocatalyst in the *n*-heptane solvent at 30 °C and 10 bar ethylene pressure [28]. In 2022, the Jian group reported a series of α-diimine nickel complexes **17** to generate UHMWPE using MMAO as a cocatalyst at ambient conditions of 30 °C and 1 bar ethylene pressure in a glass reactor [29].

**Figure 5:**
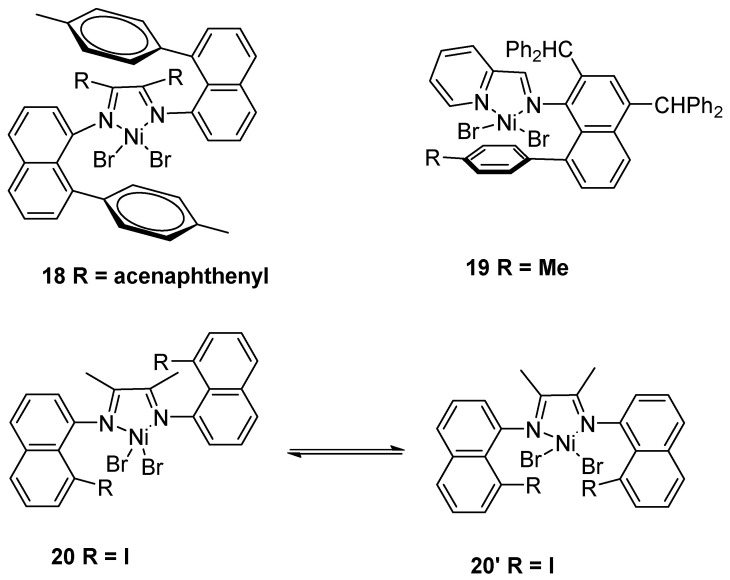
Sandwich α-diimine nickel catalysts **18**–**20** for ethylene polymerization to UHMWPE.

An innovative strategy was successfully applied in producing UHMWPE using sandwich-type structure α-diimine nickel complexes. In 2013, the Brookhart and Daugulis group discovered a new class of α-diimine-based nickel catalyst **18** with 8-aryl-1-naphthylamine substituents to produce UHMWPE using MMAO as a cocatalyst (Figure 5) [30]. Notably, the corresponding polymer was obtained with *M*n up to 1.78 × 10^6^ g·mol^−1^ at 25 °C and 27.2 bar ethylene pressure. In 2016, the Chen group achieved a unique iminopyridyl nickel complex **19** to generate UHMWPE using MAO as a cocatalyst [31]. More importantly, when the polymerization temperature was at −20 °C, the corresponding product could be generated with *M*n up to 1.43 × 10^6^ g·mol^−1^. In 2019, the Brookhart and Daugulis group reported α-diimine nickel catalyst **20** with 8-halonapthalen-1-amines moieties to prepare UHMWPE using MMAO as a cocatalyst [32]. Significantly, catalyst **20** showed the existence of *syn-* and *anti-*diastereomers in the ethylene polymerization process.

### 2.2. Nickel-Based Catalysts with N,O Ligands

**Figure 6:**
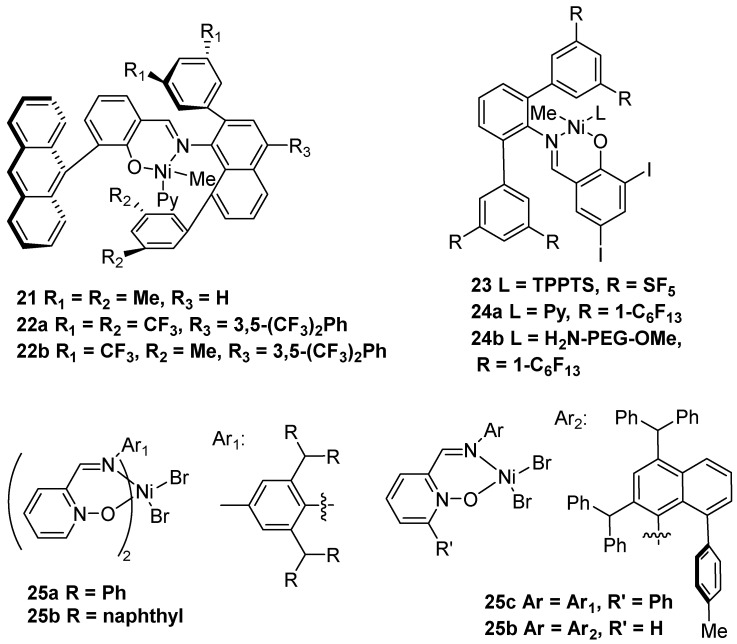
Nickel catalysts **21–25** with N,O ligands for ethylene polymerization to UHMWPE.

Apart from nickel-based catalysts with N,N′ ligands, another type of nickel catalyst with N,O ligands has living polymerization characteristics to prepare UHMWPE in polar solvents. In 2015, the Brookhart and Daugulis group achieved a neutral nickel complex with the N,O ligand to prepare branched UHMWPE in a toluene solvent without a cocatalyst (Figure 6) [33]. Interestingly, the nickel catalyst **21** showed a “quasi-living” polymerization behavior to obtain the corresponding polymer with *M*n up to 1.60 × 10^6^ g·mol^−1^. Subsequently, the Mecking group reported a similar structure of nickel complexes **22** with a salicylaldiminato ligand to generate UHMWPE in polar solvents like THF or diethyl ether [34]. Notably, catalyst **22b** could produce UHMWPE with *M*n up to 1.45 × 10^6^ g·mol^−1^ in toluene at 60 °C and 40 bar ethylene pressure.

In 2017, the Mecking group disclosed nickel complexes **23** with an SF_5_-substituted salicylaldiminato ligand to synthesize aqueous dispersions of disentangled linear UHMWPE [35]. It should be noted that the nickel catalyst with pentafluorosulfanyl substituents as well as the existence of the TPPTS ligand could accomplish the ethylene polymerization in water at 15 °C and 40 bar ethylene pressure. In addition, the same group further achieved long-lived water-stable nickel complexes **24** to produce uniform nanocrystals of UHMWPE [36]. Notably, catalyst **24b** could prepare UHMWPE with *M*n up to 3.08 × 10^6^ g·mol^−1^ in aqueous surfactant solution without cocatalyst.

In 2018, the Chen group achieved a series of bis- and monoligated 2-iminopyridine *N*-oxide nickel complexes **25** to generate UHMWPE in toluene at 20 °C and 8 bar ethylene pressure [37]. More importantly, catalyst **25c** could produce UHMWPE with *M*w up to 3.02 × 10^6^ g·mol^−1^ using low cocatalyst loading (MAO/Ni = 80).

**Figure 7:**
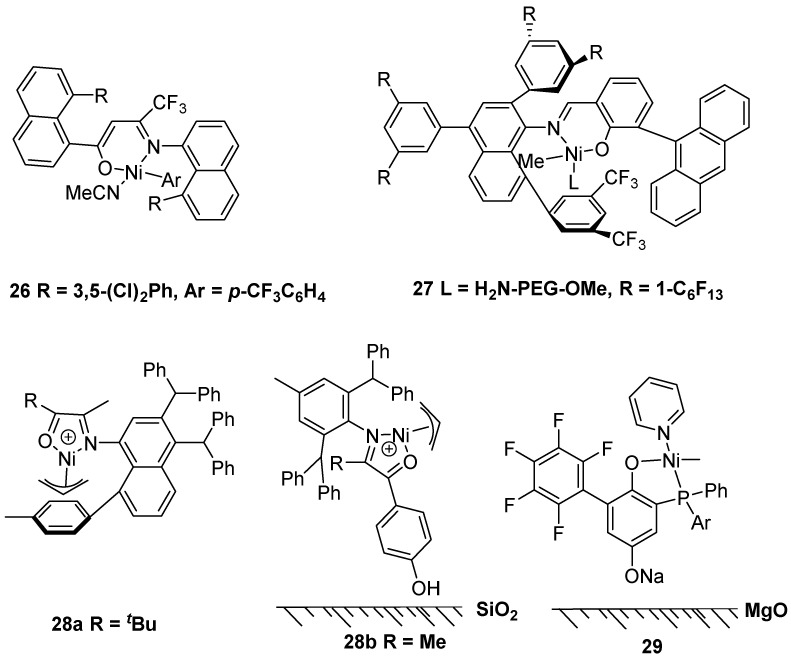
Nickel catalysts **26**–**29** with N,O ligands for ethylene polymerization to UHMWPE.

In 2020, the Brookhart and Daugulis group reported neutral nickel complex **26** with an anionic N,O- chelating ligand as a sandwich catalyst to produce branched UHMWPE with *M*n up to 4.10 × 10^6^ g·mol^−1^ in toluene without a cocatalyst (Figure 7) [38]. At the same time, the Mecking group achieved the nickel complex **27** with a salicylaldiminato ligand to generate UHMWPE through living ethylene polymerization without a cocatalyst [39]. To obtain corresponding UHMWPE, the ethylene polymerization conditions entailed 40 bar ethylene pressure and a 15 °C reaction temperature. Subsequently, the Bryliakov and Chen group nearly simultaneously achieved α-iminoketone nickel complexes **28** to afford branched UHMWPE in toluene without a cocatalyst [40,41]. More importantly, the heterogeneous catalyst **28b** with SiO_2_ supported could produce UHMWPE with *M*n up to 1.59 × 10^6^ g·mol^−1^ in heptane at 50 °C and 15 bar ethylene pressure. Recently, the same group further reported the heterogeneous catalyst **29** with MgO supported could produce UHMWPE [42].

**Figure 8:**
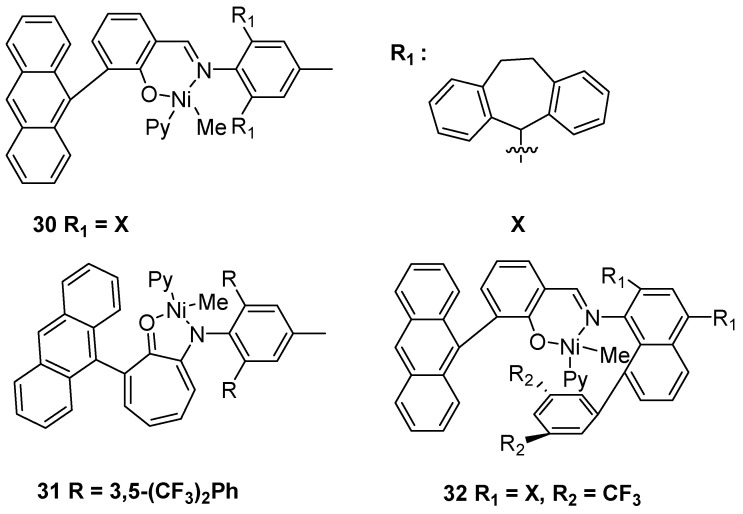
Nickel catalysts **30**–**32** with N,O ligands for ethylene polymerization to UHMWPE.

In 2021, the Jian and Mecking group reported neutral nickel complex **30** with dibenzosuberyl substituents to generate UHMWPE in toluene solvent (Figure 8) [43]. Notably, the ethylene polymerization even in tetrahydrofuran (THF) as a polar reaction solvent could prepare UHMWPE with *M*n = 1.41 × 10^6^ g·mol^−1^ at 30 °C and 40 bar ethylene pressure. In 2022, the Jian and Kang group achieved neutral anilinotropone nickel complex **31** to produce linear UHMWPE in toluene and 8 bar ethylene pressure [44]. It should be noted that the nickel catalyst with a salicylaldiminato ligand instead of an anilinotropone structure could not obtain the UHMWPE under otherwise identical conditions. At the same time, the Jian group discovered a neutral sandwich-like salicylaldiminato nickel catalyst **32** to synthesize UHMWPE in polar and non-polar solvents [45]. Mainly, THF as a polar solvent could produce linear UHMWPE with a positive solvent effect in a living ethylene polymerization manner.

### 2.3. Nickel-Based Catalysts with P Ligands

**Figure 9:**
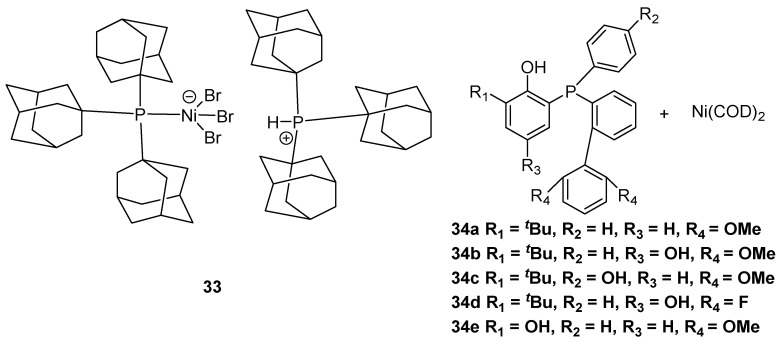
Nickel catalysts **33**–**34** with P ligands for ethylene polymerization to UHMWPE.

In addition, nickel-based catalysts with P ligands could obtain functionalized UHMWPE through good copolymerization ability with polar monomers. In 2019, the Daugulis group disclosed the tri-1-adamantylphosphine-nickel complex **33** to produce UHMWPE using polymethylaluminoxane (PMAO) as a cocatalyst (Figure 9) [46]. It should be noted that this catalytic system afforded nearly linear UHMWPE with *M*n up to 1.68 × 10^6^ g·mol^−1^ at 10 °C in toluene. Recently, the Chen and Zou group developed a series of phosphino-phenolate ligands **34** that they can combine with Ni(COD)_2_ in situ to prepare UHMWPE using the precipitation polymerization strategy [47]. More interestingly, these nickel catalysts (in situ) can catalyze precipitation copolymerization of ethylene and polar monomers to obtain functionalized UHMWPE with *M*n up to 1.57 × 10^6^ g·mol^−1^ at 20 °C and 30 bar ethylene pressure in toluene.

### 2.4. Palladium-Based Catalysts with N,N′ Ligands

**Figure 10:**
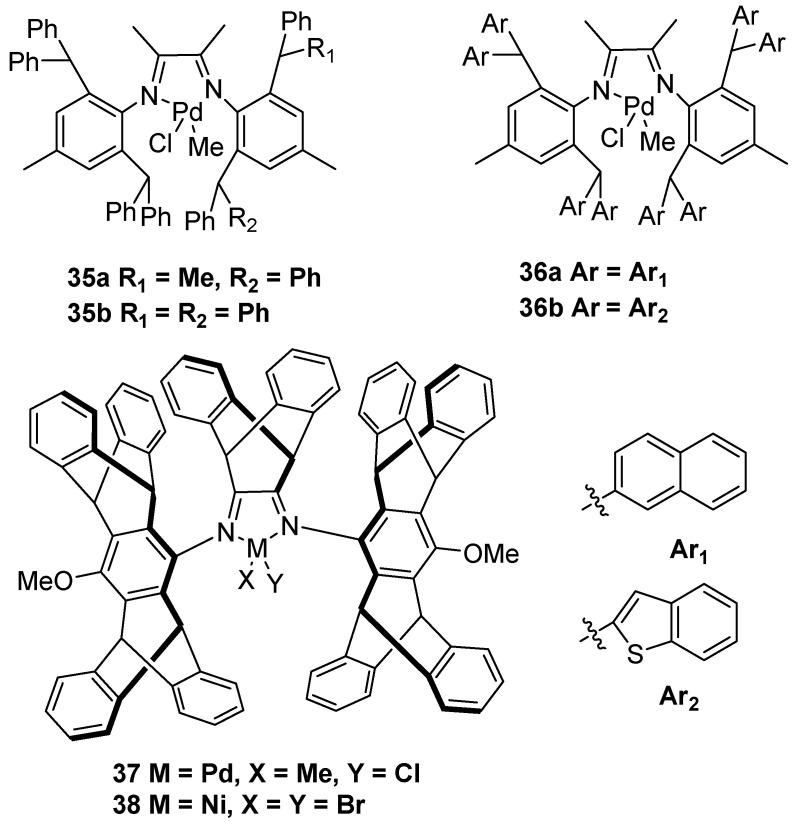
Palladium catalysts **35–38** with N,N′ ligands for ethylene polymerization to UHMWPE.

Particularly, palladium-based catalysts with N,N′ ligands could be potential catalysts in the preparation of UHMWPE by industrially supported technology. In 2016, the Chen group achieved α-diimine palladium complexes **35** to produce UHMWPE in the presence of 1.2 equiv of sodium tetrakis(3,5-bis(trifluoromethyl)phenyl)borate (NaBAF) (Figure 10) [48]. It should be noted that catalyst **35b** could obtain UHMWPE with *M*n up to 1.65 × 10^6^ g·mol^−1^ at 40 °C and 8 bar ethylene pressure. Subsequently, the same group disclosed analogous structure palladium complexes **36** with α-diimine ligands to generate UHMWPE [49]. More importantly, catalysts **36a** and **36b** could copolymerize ethylene with polar monomers to afford UHMWPE. Interestingly, the authors discovered that these α-diimine palladium complexes could be suitable for gas-phase and slurry-phase ethylene polymerization to prepare branched UHMWPE using a self-supporting strategy [50]. These breakthrough achievements provided a possible method to produce UHMWPE through gas-phase and slurry-phase polymerization techniques in the polyolefin industry. In 2020, the Jian and Mecking group synthesized a novel α-diimine palladium complex **37** with a bulky dibenzobarrelene backbone and axial pentiptycenyl substituents to generate branched UHMWPE using NaBAF as an additive [51]. Notably, the palladium catalyst could generate corresponding UHMWPE with *M*w up to 1.57 × 10^6^ g·mol^−1^ at 50 °C and 8 bar ethylene pressure. Recently, the same group further achieved α-diimine nickel catalyst **38** with analogous structure ligands to afford branched UHMWPE [52].

### 2.5. Iron and Cobalt-Based Catalysts with N,N′ Ligands

**Figure 11:**
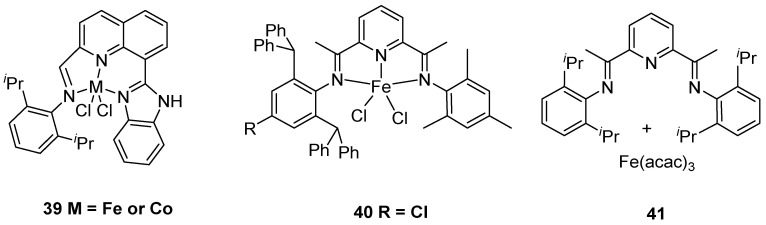
Iron and cobalt catalysts **39**–**41** with N,N′ ligands for ethylene polymerization to UHMWPE.

Iron and cobalt complexes could also be applied in ethylene polymerization to generate UHMWPE (Figure 11). Interestingly, this type of catalyst could produce weakly entangled UHMWPE. In 2011, the Redshaw and Sun group reported iron and cobalt complexes **39** with 2-(1-(arylimino)methyl)-8-(1H-benzimidazol-2-yl)quinoline ligands to produce UHMWPE at 60 °C using MAO as a cocatalyst [53]. More importantly, the cobalt catalyst shows excellent thermal stability at 80 °C to generate UHMWPE with *M*w up to 1.23 × 10^6^ g·mol^−1^. Compared with the iron catalyst, the corresponding cobalt complex could tolerate higher temperatures in ethylene polymerization to generate UHMWPE.

In 2012, the same group further achieved iron complex **40** with 2-[1-(2,6-dibenzhydryl-4-chlorophenylimino)ethyl]-6-[1-(arylimino)ethyl]pyridine ligand to generate UHMWPE using MMAO as a cocatalyst [54]. It should be noted that the iron catalyst could afford corresponding UHMWPE with *M*w up to 1.20 × 10^6^ g·mol^−1^ in toluene at 20 °C and 10 bar ethylene pressure. In 2015, the Li group disclosed that the 2,6-bis [1-(2-isopropylanilinoethyl)] pyridyl ligand **41** can combine with iron acetylacetonate (Fe(acac)_3_) in situ to prepare weakly entangled UHMWPE [55]. Notably, the synthesized corresponding polymer showed a significantly high melting point. Compared with the iron catalyst **40** by the Redshaw and Sun group, the Fe(acac)_3_ with a corresponding ligand in situ to prepare the iron complex **41** is an easier synthesis route and provides more flexible ligand structures.

## 3. Conclusions and Perspective

In this review, we summarized the significant progress in the area of late transition metal catalysts for ethylene polymerization to produce UHMWPE in the past decade. Several classes of late transition metal catalysts such as α-diimine nickel catalysts, salicylaldiminato nickel catalysts, nickel-based catalysts with P ligands, palladium-based catalysts with N,N′ ligands, as well as iron and cobalt-based catalysts with N,N′ ligands were discussed in detail. These late transition metal catalysts allow the generation of linear or branched UHMWPE with narrow molecular weight distribution, as well as incorporate polar monomers to obtain functionalized polyolefin. More importantly, some nickel catalytic systems could prepare UHMWPE in polar solvents like water and diethyl ether. Despite these advances in academic research, there has been no successful commercialization example of late transition metal-based olefin polymerization catalysts. Further refinement of catalyst structure and performance, cost management, molecular weight regulation, as well as industrial scale reactions still entail more in-depth research by chemists. We believe that this area will make a significant technical breakthrough soon.

## Data Availability

All available data during this study are included in this article.
